# Necroptosis-Associated lncRNA Prognostic Model and Clustering Analysis: Prognosis Prediction and Tumor-Infiltrating Lymphocytes in Breast Cancer

**DOI:** 10.1155/2022/7099930

**Published:** 2022-04-27

**Authors:** Shigui Tao, Kunlin Tao, Xiaoyong Cai

**Affiliations:** ^1^The General Surgery, The Second Affiliated Hospital of Guangxi Medical University, No. 166, Daxuedonglu Road, Nanning, Guangxi, China; ^2^The People's Hospital, Guiping, Guangxi, China

## Abstract

Necroptosis plays an important role in tumor genesis and progression. This study aims to identify necroptosis-related lncRNAs (NR-lncRNAs) in breast cancer (BC), and their prognostic value and relationship with the tumor immune environment (TIE) through bioinformatics. *Methods*. A total of 67 necroptosis-related genes (NRGs) are retrieved, and 13 prognostically relevant NR-lncRNAs are identified by co-expression and Univariate Cox regression analyses. After unsupervised clustering analysis, the patients are classified into three clusters, and their survival and immune infiltration are compared. Lasso regression analysis is conducted to construct a prognostic model using eight lncRNAs (USP30-AS1, AC097662.1, AC007686.3, AL133467.1, AP006284.1, NDUFA6-DT, LINC01871, AL135818.1). The model is validated by Kaplan-Meier survival analysis, Multivariate Cox regression analysis, and receiver-operating characteristic (ROC) curves. Correlation analysis is useful to identify associations between risk scores and clinicopathological features. GSEA, drug prediction, and immune checkpoints analysis are further used to differentiate between the risk groups. *Results*. The C3 cluster has longer overall survival (OS) and the highest immune score, indicative of an immunologically hot tumor that may be sensitive to immunotherapy. Furthermore, the OS is significantly higher in the low-risk group, even after dividing the patients into subgroups with different clinical characteristics. The area under the ROC curve (AUC) for 1-, 3-, and 5-year survival in the training set are 0.761, 0.734, and 0.664, respectively, which indicate the moderate predictive performance of the model. *Conclusion*. NR-lncRNAs can predict the prognosis of BC, distinguish between hot and cold tumors, and are potential predictive markers of the immunotherapy response.

## 1. Introduction

Breast cancer (BC) is the most prevalent malignancy in women and has surpassed lung cancer in terms of incidence worldwide [1]. The current therapeutic approaches have increased the 5-year survival rate of BC patients to 90% in developed countries, although the rate varies greatly by region [2]. Although long considered non-immunogenic or weakly immunogenic tumors, there is a renewed interest in developing immunotherapies against BC due to the encouraging outcomes of other tumors such as melanoma and non-small cell lung cancer. In fact, immune checkpoint inhibitors have been effective against BC in clinical practice. However, only a fraction of cancer patients benefit from immunotherapy due to considerable heterogeneity in treatment sensitivity and side effects [3]. Furthermore, there is a paucity of reliable biomarkers for monitoring the effects of immunotherapy. Thus, it is crucial to identify novel biomarkers to predict the efficacy of immunotherapy and devise individualized therapy plans for cancer patients [4].

Recent studies suggest that some pathological subgroups of breast tumors are rich in tumor-infiltrating lymphocytes (TILs), which serve as a reliable prognostic biomarker of BC. For every 10% increase in TILs, the risk of recurrence and death decreases by varying degrees [5–8]. In particular, the infiltration of cytotoxic CD8+ T lymphocytes, CD4+ T lymphocytes, and tumor-associated macrophages (TAMs) predicts favorable outcomes [9, 10]. Since chemotherapeutic drugs can enhance the anti-tumor immune response by clearing the immunosuppressive cells or coaxing the tumor cells to release neoantigens [11, 12], higher levels of TILs can improve tumor response to immune checkpoint inhibitors [4].

Necroptosis is a programmed form of necrosis that occurs in a caspase-independent manner and partly via the apoptosis pathways [13]. Apoptosis inhibition is one of the mechanisms employed by tumor cells to acquire drug resistance, which leads to chemotherapy failure [14]. Induction of necroptosis is a potential therapeutic alternative given its crucial role in tumorigenesis, metastasis, and anti-tumor immunity [15]. Furthermore, the necroptosis signaling factor RIPK3 is known to regulate the function of dendritic cells (DCs) and natural killer cells (NKs), and Fas-associated death domain (FADD) can restrain T cell-mediated necroptotic signaling [16–18]. Necroptosis also initiates an adaptive immune response by liberating damage-associated molecular patterns (DAMPs) from the dying tumor cells, prompting the phagocytes to release pro-inflammatory cytokines [19, 20]. However, there are reports that necroptosis can promote tumor progression by recruiting pro-tumorigenic inflammatory cells [21]. Therefore, the exact role of necroptosis in tumor progression and immune response needs further investigation.

The long non-coding RNAs (lncRNAs) regulate the expression of genes involved in cell cycle control and signal transduction pathways, thereby affecting tumor cell proliferation, apoptosis, metastasis, and invasion [22, 23]. In addition, several lncRNAs have been identified that are related to the functional regulation of TILs [24]. For instance, low expression of lncRNA BM466146 in BC cells allows more competitive endogenous RNAs (ceRNAs) to bind to hsa-miR-224-3p, which upregulates CXCL-13 and eventually activates the cytotoxic CD8+ T cells [25]. Likewise, silencing the lncRNA SNHG1 inhibits the differentiation of Treg cells and immune escape of BC cells by promoting miR-448 expression and reducing indoleamine 2, 3-dioxygenase (IDO) levels [26]. In this study, we identified the necroptosis-related lncRNAs (NR-lncRNAs) in BC and compared the immune signatures of patients classified based on NR-lncRNAs to screen ideal candidates for immunotherapy and improve prognosis.

## 2. Materials and Methods

### 2.1. Data Extraction

Sixty-seven necroptosis-related genes (NRGs) were obtained by reviewing relevant literature and are listed in Appendix 1 [27]. The survival and transcriptomic (FPKM format) data of 1078 BC samples was retrieved from the TCGA database (https://portal.gdc.cancer.gov/projects/TCGA-BRCA). The data were extracted and collated using Strawberry Perl.

### 2.2. Identification of NR-lncRNAs

The transcriptomic data was divided into lncRNA and mRNA data using Strawberry Perl software and annotation files. The expression matrix data of the 67 NRGs were extracted using the limma R package with correlation factor =0.4 and *p* value =0.001 as the thresholds. A total of 1470 NR-lncRNAs were identified by co-expression analysis, organized into expression matrices, and merged with the survival data. Univariate Cox regression analysis was conducted using the survival R package with *p* value =0.01 as the threshold, and 13 prognostically relevant lncRNAs (PR-lncRNAs) were identified and used to draw the corresponding forest plots. The differentially expressed PR-lncRNAs between tumor and normal samples were identified using the limma R package (∗*p* < 0.05, ∗∗*p* < 0.01, ∗∗∗*p* < 0.001).

### 2.3. Immune Infiltration Analysis

The potential subgroups of BC based on PR-lncRNAs were identified using the ConsensusClusterPlus R package. The survivals of patients in the different subgroups were compared using the Kaplan-Meier method. The correlation between the expression of PR-lncRNAs and that of PD-L1 and CTLA-4 was also analyzed. The differential expression of PR-lncRNAs between subgroups and their correlation were analyzed using the limma R package. Immune cell infiltration in the different subgroups was evaluated using the CIBERSORT algorithm, and the ESTIMATE algorithm was used to calculate the Immune, Stromal, and ESTIMATE scores of each sample to assess tumor purity.

### 2.4. Construction and Validation of NR-lncRNAs Prognostic Model

The samples were divided into the training set and the validation set at a 1 : 1 ratio using the caret R package. The clinical characteristics of patients in the two data sets were compared by the chi-square test. The PR-lncRNAs were selected for Lasso regression analysis to avoid overfitting and remove closely related genes. In contrast, the minor penalty term (*λ*) was chosen using cross-validation. The optimal model for predictive performance was then selected using the glmnet R package, and constructed based on eight PR-lncRNAs. The equation for the risk score is shown below:

(1) $$\begin{array}{c} \text{Risk score}=\overset{n}{\underset{i=1}{\sum }}\;{\text{Coef}}_{i}*x_{i}, \end{array}$$

where Coef_*i*_ represents the risk coefficient and *x*_*i*_ represents the expression of each lncRNA. Patients were classified into high-risk and low-risk groups based on the median risk score. The R packages survival, survminer, and timeROC were used for survival analysis and model evaluation. Kaplan-Meier survival curves and ROC curves for 1-, 3- and 5-year survival were plotted for the training and validation sets.

The independent prognostic value of the model was confirmed by Univariate and Multivariate Cox regression analyses, and the survival rates in different subgroups classified on the basis of age and tumor stages were compared. The differences in risk scores between the above subgroups, and the differential expression of the eight PR-lncRNAs between high- and low-risk groups were analyzed using R packages limma and ggpubr.

### 2.5. Comparison of High- and Low-Risk Groups

The correlation between immune cells and risk score was analyzed using R packages limma, ggplot2, ggpubr, and ggExtra. A total of 47 immune checkpoints were obtained by scanning relevant literature (in Appendix 2). Differential expression analysis of immune checkpoint genes between risk subgroups was performed using the limma package (∗*p* < 0.05, ∗∗*p* < 0.01, ∗∗∗*p* < 0.001). The pRRophetic package was used to predict the IC50 of anti-tumor drugs to identify responsive subgroups. GSEA 4.1.0 was used to compare gene set functions between the high- and low-risk groups with *p* < 0.05 and FDR<0.25 as the criteria.

## 3. Results

### 3.1. Identification of Prognostically Relevant NR-lncRNAs in BC

**Figure 1:**
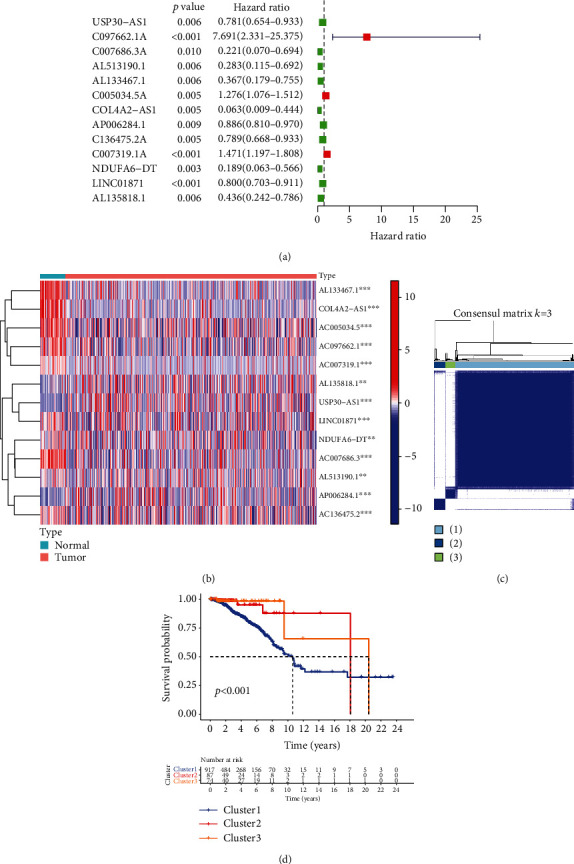
Extraction and unsupervised consensus clustering analysis of 13 prognosis-related lncRNAs. (a). Forest plot of 13 prognosis-related lncRNAs based on Univariate Cox regression analysis. (b). Heat map showing the expression of 13 prognosis-related lncRNAs between tumor and normal samples. (c). Consensus matrix *k* =3. (d). Kaplan-Meier survival curves for C1, C2, and C3 clusters.

We extracted 67 NRGs from the mRNA expressionmatrix of BC samples from the Cancer Genome Atlas and identified 1470 NR-lncRNAs through co-expression analysis. Furthermore, 13 of these NR-lncRNAs were prognostically significant, including the pro-oncogenic AC097662.1, AC005034.5, and AC007319.1 (HR>1), and the anti-oncogenic USP30-AS1, AC007686.3, AL513190.1, AL133467.1, COL4A2-AS1, AP006284.1, AC136475.2, NDUFA6-DT, LINC01871, and AL135818.1 (HR<1; *p* < 0.01) (Figure 1(a)). Each of the above lncRNAs was differentially expressed between the tumor and normal samples (Figure 1(b), Figure S1).

### 3.2. The PR-lncRNAs of BC Correlate with Immune Checkpoints and TILs

**Figure 2:**
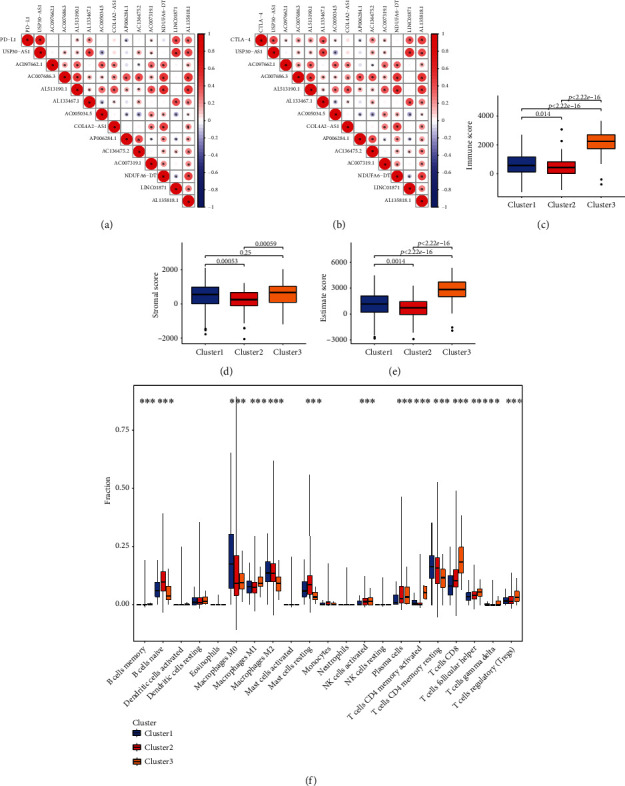
Correlation of prognosis-related lncRNAs with the tumor immune landscape. Correlation of 13 prognosis-related lncRNAs with (a) PD-L1 expression, (b) CTLA-4 expression, (c) Immune scores, (d) Stroma scores, (e) ESTIMATE scores, and (f) Immune cell infiltration in the C1, C2, and C3 clusters.

The patients were regrouped into three clusters on the basis of the PR-lncRNAs, of which C1 showed lower overall survival (OS) rates than that of C2 or C3, indicating that the PR-lncRNAs are strongly associated with the prognosis of BC (Figures 1(c) and 1(d)). In addition, all PR-lncRNAs except AC097662.1 and COL4A2-AS1 showed significant differences in their expression level between at least two patient clusters (Figure S2, S3). We further analyzed the correlation between the PR-lncRNAs and immune checkpoints PD-L1 and CTLA-4. PD-L1 was positively correlated with USP30-AS1, AL513190.1, AL133467.1, AC007319.1, LINC01871, and AL135818.1, and negatively with AP006284.1. CTLA-4 showed positive correlation with USP30-AS1, AC097662.1, AC007686.3, AL513190.1, AL133467.1, AC005034.5, AC136475.2, AC007319.1, LINC01871, and AL135818.1, and negative correlation with AP006284.1 (Figures 2(a) and 2(b)).

The C3 cluster had the highest Immune and ESTIMATE scores, followed by C2 and C1 (Figures 2(c)–2(e)). Furthermore, the anti-tumor immune cell populations such as CD4+ activated memory T cells, CD8+ T cells, follicular helper T cells, memory B cells, M1 macrophages, and NK cells were more abundant in the C2 and C3 clusters. In contrast, the infiltration of the CD4+ resting memory T cells, naïve B cells, M0 and M2 macrophages, resting mast cells, and resting NK cells was either higher in the C1 cluster or similar among all clusters. Monocytes and neutrophils were equally abundant in all three clusters (Figure 2(f), Figure S4).

### 3.3. Construction and Validation of Risk Model

**Figure 3:**
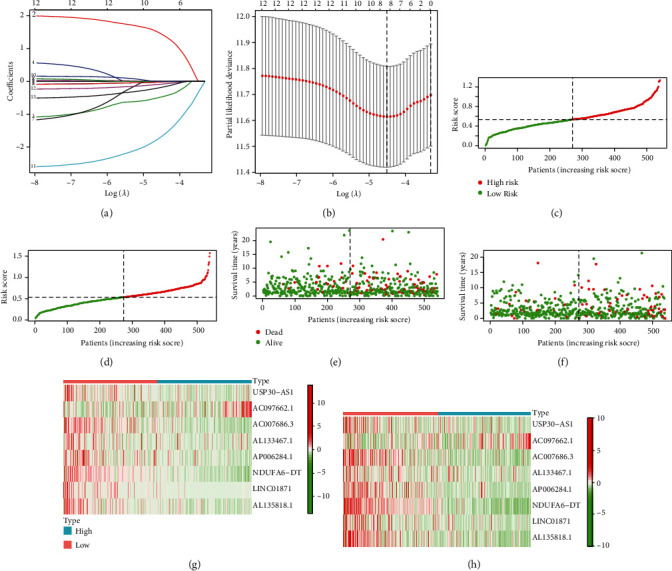
Construction of risk model. (a) and (b) Eight lncRNAs identified by the Lasso regression analysis. (c) and (d) Risk scores of patients in the (c) training and (d) validation sets. (e) and (f) Survival status of patients in the (e) training and (f) validation sets. (g) and (h) Expression levels of 8 lncRNAs in the high- and low-risk groups in (g) training and (h) validation sets.

Lasso regression analysis was performed with the 13 PR-lncRNAs, and USP30-AS1, AC097662.1, AC007686.3, AL133467.1, AP006284.1, NDUFA6-DT, LINC01871, and AL135818.1 were used to construct the prognostic model (Figures 3(a) and 3(b)). The patients were divided into the training and validation sets, which were comparable since the clinicopathological features did not show significant differences (Table 1). The risk score, survival status, and expression of the 13 PR-lncRNAs in both data sets are shown in Figure 3 (training set - c, e, and g; validation - d, f, and h). The risk score was calculated as follows: USP30-AS1 expression ∗ (-0.0051) + AC097662.1 expression ∗ (1.4470) + AC007686.3 expression∗ (-0.4771) + AL133467.1 expression ∗ (-0.0146) + AP006284.1 expression ∗ (-0.0188) + NDUFA6-DT expression ∗ (-1.6889) + LINC01871 expression∗ (-0.0852) + AL135818.1 expression ∗ (-0.1846).

**Figure 4:**
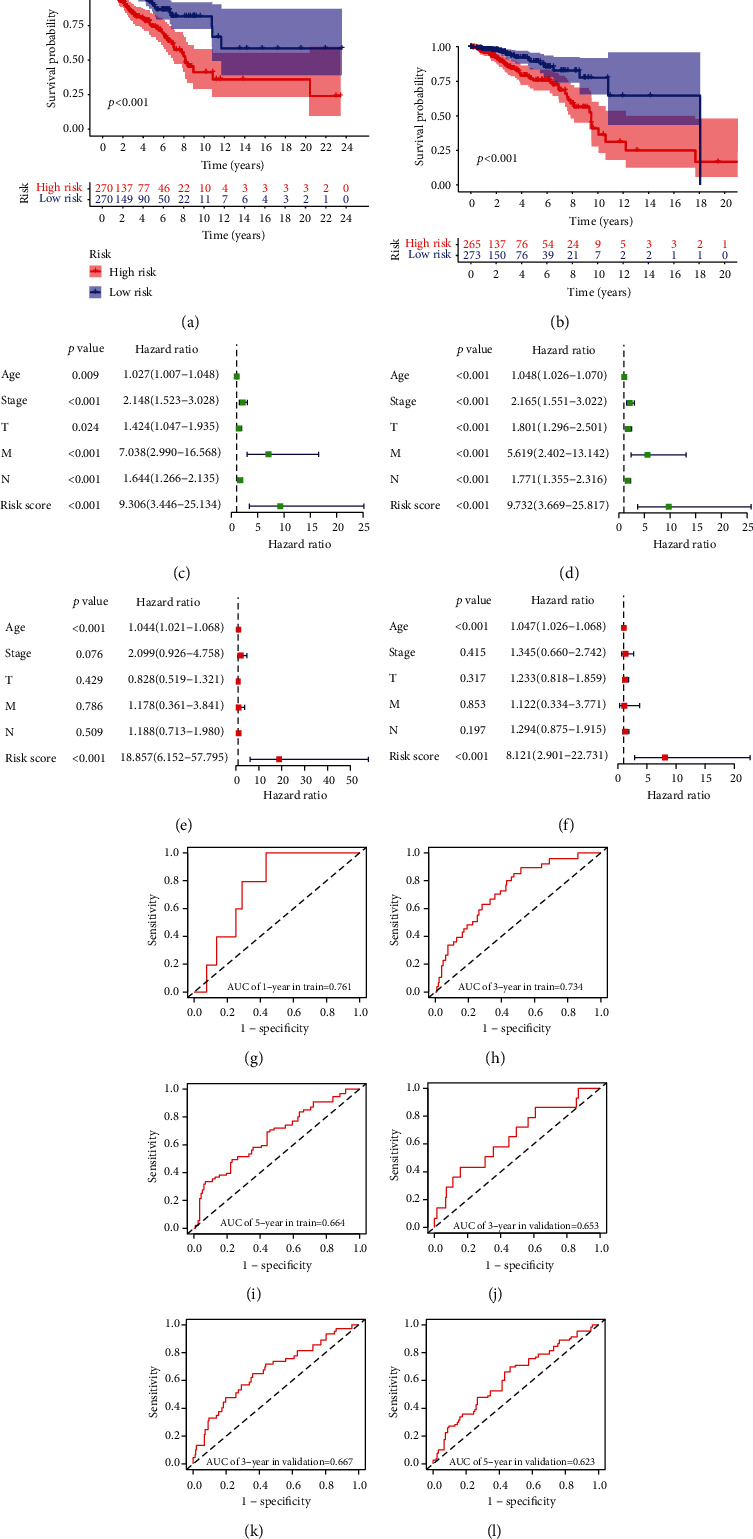
Validation of risk model. (a) and (b) Kaplan-Meier survival curves of high- and low-risk groups in the (a) training and (b) validation sets. (c) and (d) Univariate Cox regression analysis for risk score and clinicopathological features in the (c) training and (d) validation sets. (e) and (f) Multivariate Cox regression analysis of the risk score in (e) training and (f) validation sets. (g)–(l) The AUC of 1-, 3-, and 5-year OS in the (g)–(i) training and (j)–(l) validation sets.

**Figure 5:**
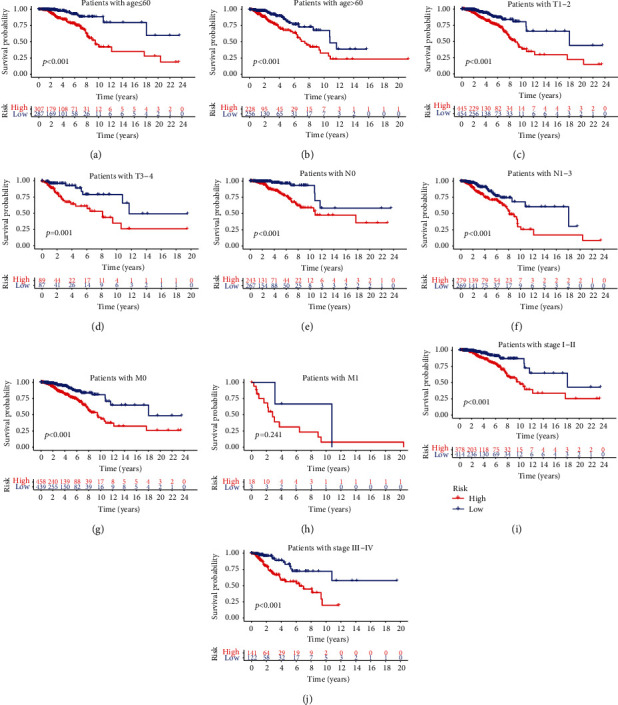
Kaplan-Meier survival curves of the high- and low-risk patients in the <60 years, >60 years, Stage I-II, Stage III-IV, T1-2, T3-4, N0, N1, M0, and M1 subgroups.

Kaplan-Meier survival analysis of both training and validation sets revealed that the high-risk group had significantly worse OS than the low-risk group (Figures 4(a) and 4(b)). Furthermore, the area under the ROC curve (AUC) of 1-, 3-, and 5-year OS for the training set was 0.761, 0.734, and 0.664, and that for the validation set was 0.653, 0.667, and 0.623, respectively (Figures 4(g)–4(l)), which indicated the predictive value of the risk score. Multivariate Cox regression analysis further showed that the risk score was an independent predictor of worse survival as opposed to age and tumor stage. Therefore, the risk score-based model may be more reliable than clinicopathological factors for predicting the patient prognosis (Figures 4(c)–4(f)). We also compared the OS of the high- and low-risk groups that were divided into subgroups of age (<60 years and >60 years) and tumor stage (I-II, III-IV, T1-2, T3-4, N0, N1, M0, and M1), and found that the high-risk group had shorter OS regardless of the age and tumor stage (Figures 5(a)–5(j)). Taken together, the lncRNA-based risk model can be applied to elderly and young, as well as early to advanced BC patients.

### 3.4. The Necroptosis Risk Score Correlated with Tumor Immune Status

**Figure 6:**
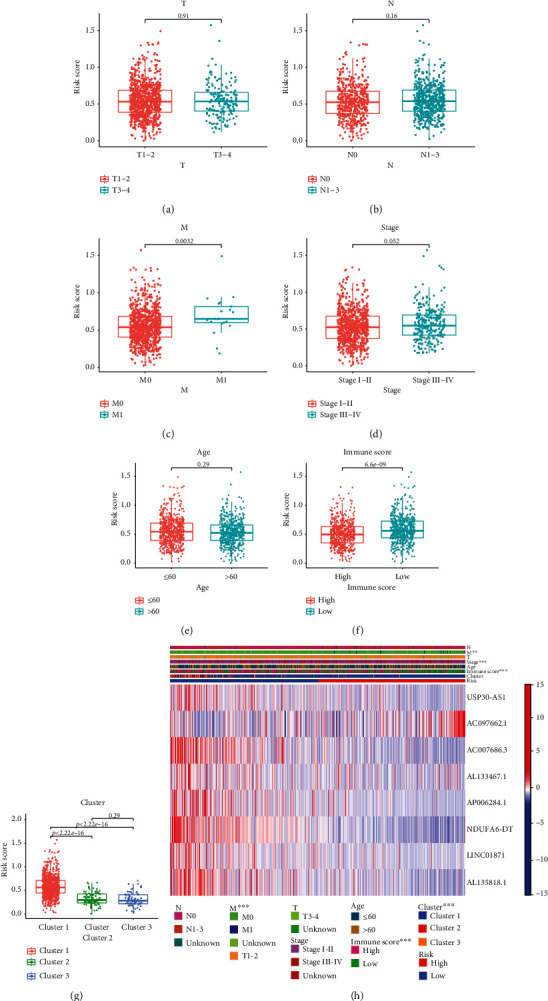
The correlation between risk score and clinicopathological factors, Immune score and patient clusters. Distribution of risk scores in (a) T1-2 and T3-4, (b) N0 and N1-3, (c) M0 and M1, (e) different age groups, (f) Immune score groups, and (g) patient clusters. (h) Heat map of 8 lncRNAs and clinicopathological factors.

**Figure 7:**
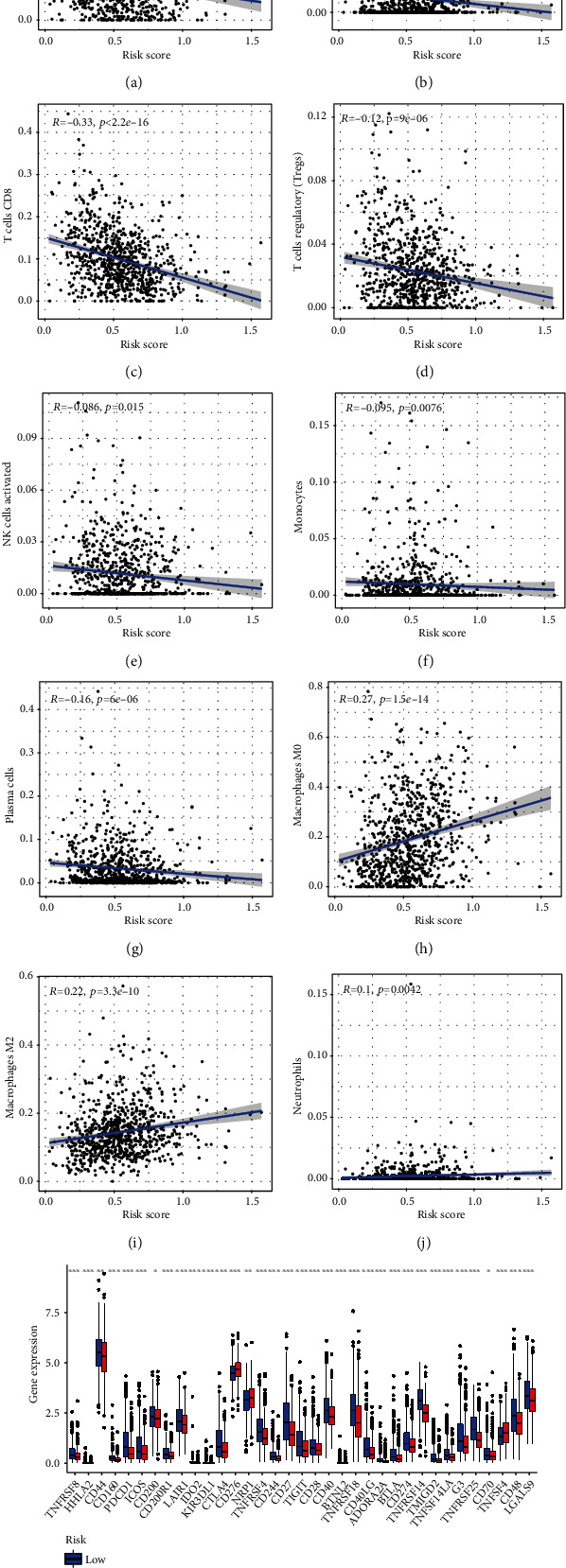
Immune cells infiltration and checkpoints in the high- and low-risk groups. (a)–(g) Abundant immune cell types in the low-risk group. (h)–(j) Abundant immune cell types in the high-risk group. (k) Expression of 47 immune checkpoints in high- and low-risk groups.

The risk score was significantly different between the M0 vs. M1, C1 vs. C2 or C3, and high- vs. low-immune infiltration groups. Patients without distant metastases, in clusters C2 and C3, or with high-immune scores usually presented lower risk scores and better survival prognoses (Figures 6(a)–6(g)). Furthermore, AC097662.1 was highly expressed in the high-risk group, while the other lncRNAs showed the opposite trend corresponding to their risk coefficients in the prognostic model (Figure 6(h)). These findings indicate that necroptosis is associated with the tumor immune status and distant metastasis. The predominant tumor-infiltrating immune cells in the low-risk group were naïve B cells, monocytes, activated NK cells, plasma cells, CD4+ activated memory T cells, and CD8+ T cells. In contrast, high infiltration of M0 and M2 macrophages and neutrophils was observed in the high-risk group (Figures 7(a)–7(j)). The immune cells enriched in the low-risk group were essentially the same as observed for the C2 or C3 clusters, whereas that in the high-risk group corresponded to the immune cell profile of the C1 cluster. Taken together, the risk score can effectively predict the infiltrating immune cells in the breast tumor microenvironment.

### 3.5. The Necroptosis Risk Score Can Predict Response to Immune Checkpoint Inhibitors

We next compared the expression of immunotherapy-related genes and the IC50 of therapeutic agents between the two risk groups. Immune checkpoints including PD-L1 (CD274), CD28 (CTLA-4 homolog), and CTLA-4, which are closely associated with BC immunotherapy, were enriched in the low-risk group (Figure 7(k)), indicating that these patients may be more sensitive to immunotherapy. In addition, therapeutic agents except A.443654, A.770041, AZD.0530, Bicalutamide, BMS.708163, BMS.754807, BX.795, CMK, Erlotinib, GNF.2 JNJ.26854165, KIN001.135, and Lapatinib had lower IC50 in the low-risk group (Figure S5, S6). To summarize, patients in the C2 and C3 clusters with higher immunogenicity, higher sensitivity to immunotherapy, and lower risk score had better survival prognoses, whereas the C1 cluster exhibited the opposite trend. Therefore, the necroptosis-based risk score can be used to distinguish between the immunologically “cold” and “hot” breast tumors to screen for patients that may benefit from immunotherapy.

### 3.6. GSEA between High- and Low-Risk Groups

**Figure 8:**
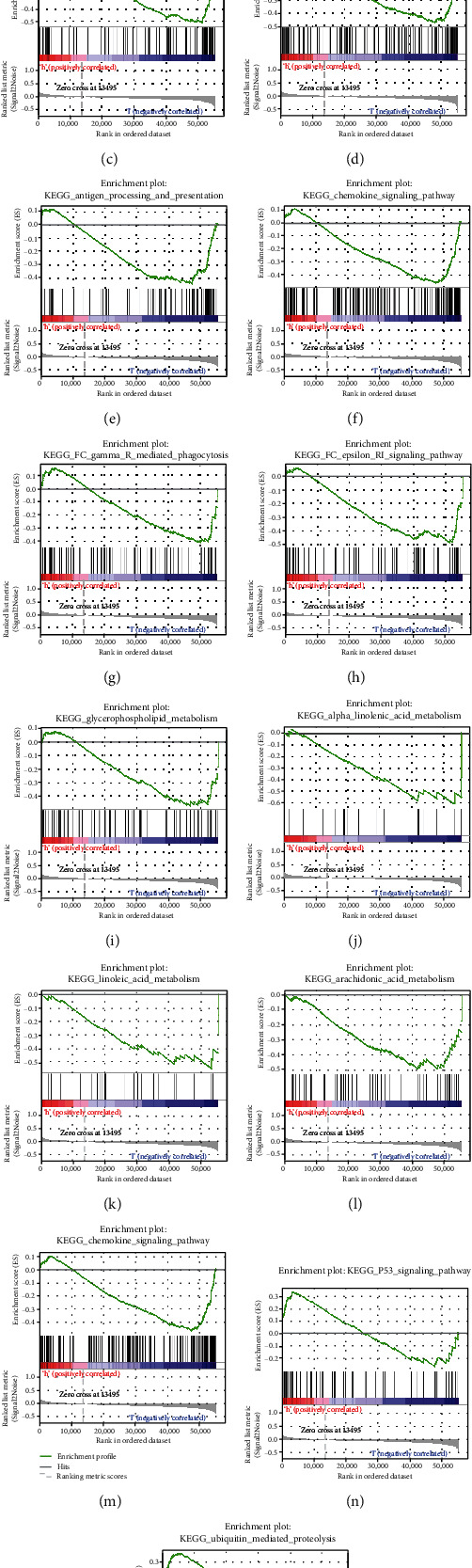
Enrichment plots of apoptosis, immune associated, lipid oxidation metabolism, and chemokine signaling gene sets from GSEA. (a) KEGG_APOPTOSIS, (b) KEGG_B_CELL_RECEPTOR_SIGNALING_PATHWAY, (c) KEGG_T_CELL_RECEPTOR_SIGNALING_PATHWAY, (d) KEGG_NATURAL_KILLER_CELL_MEDIATED_CYTOTOXICITY, (e) KEGG_ANTIGEN_PROCESSING_AND_PRESENTATION, (f) KEGG_CHEMOKINE_SIGNALING_PATHWAY, (g) KEGG_FC_GAMMA_R_MEDIATED_PHAGOCYTOSIS, (h) KEGG_FC_EPSILON_RI_SIGNALING_PATHWAY, (i) KEGG_GLYCEROPHOSPHOLIPID_METABOLISM, (j) KEGG_ALPHA_LINOLENIC_ACID_METABOLISM, (k) KEGG_LINOLEIC_ACID_METABOLISM, (l) KEGG_ARACHIDONIC_ACID_METABOLISM, (m) KEGG_CHEMOKINE_SIGNALING_PATHWAY, (n) KEGG_P53_SIGNALING_PATHWAYND, (o) KEGG_UBIQUITIN_MEDIATED_PROTEOLYSIS.

GSEA showed that the gene sets associated with apoptosis, immune function, lipid oxidation metabolism, and chemokine signaling, including apoptosis, T cell receptor signaling, B cell receptor signaling, natural killer cell-mediated cytotoxicity, antigen processing and presentation, chemokine signaling, Fc epsilon RI signaling, Fc gamma receptor-mediated phagocytosis, glycerophospholipid metabolism, alpha linolenic acid metabolism, linoleic acid metabolism, arachidonic acid metabolism, and chemokine signaling, were significantly enriched in the low-risk group (Figures 8(a)–8(m)). In the high-risk group, the p53 signaling pathway and necroptosis regulation-related gene sets were enriched, namely, the p53 signaling and ubiquitin-mediated proteolysis (Figures 8(n) and 8(o)). The enrichment in gene sets of immune-related functions in the low-risk group was consistent with abundant immune cell infiltration in the tumor microenvironment.

## 4. Discussion

### 4.1. Unsupervised Consensus Clustering Analysis

BC is highly heterogeneous in terms of tumor morphology, prognosis, and treatment response. Although receiving maximum existing therapeutic regimens, there are 20% of patients still die, and 85% of the patients do not respond to conventional chemotherapy. BC is currently classified into pathological subtypes based on estrogen receptor (ER), progesterone receptor (PR), and human epidermal growth factor 2 (HERT2), which cannot predict the response of individual patients to precision treatment regimens [28, 29]. To this end, we developed a model to predict the immunotherapy response of breast tumors based on necroptosis-related lncRNAs. Unsupervised consensus clustering analysis identified 13 PR-lncRNAs, and the patients were accordingly divided into three clusters with distinct necroptosis profiles. The C2 and C3 clusters showed higher survival rates than the C1 cluster, which led us to analyze their correlation with immune characteristics and model risk scores.

### 4.2. Construction and Validation of Risk Model

We further screened eight lncRNAs through Univariate Cox and Lasso regression analyses to construct a prognostic model, of which some have been previously associated with tumor development or prognosis. For instance, AL133467.1 is associated with a favorable immune landscape in ovarian tumors, which correlates with a good prognosis [30]. In our study, AL133467.1 was found to be beneficial for the survival of BC patients. LINC01871 is an established protective factor in BC and is associated with autophagy, ferroptosis, and tumor stem cells [31–33]. This was consistent with our findings, which led us to hypothesize that the NRGs co-expressed with LINC01871 may promote cancer cell death by necroptosis. USP30-AS1 is an intra-mitochondrial lncRNA that inhibits mitophagy and promotes mitochondrial dysfunction and oncogenic progression [34]. In addition, USP30-AS1 increases the oncogenicity of cervical cancer cells by upregulating PTP4A1 through the USP30-AS1/miR-299-3p/PTP4A1 network [35]. Likewise, USP30-AS1 was identified as an oncogene in BC, and could be a potential therapeutic target. The risk score of the lncRNA-based model was an independent predictor of poor prognosis after adjusting for age, tumor stage, and TNM stage. Furthermore, ROC curves proved the predictive performance of the model, and the survival analysis of high- and low-risk groups demarcated on the basis of age and tumor stage indicated that the model is broadly applicable to the BC patient population.

### 4.3. Association of Risk Score with Clinicopathological Factors, Clusters, and Immune Infiltration

We observed that the risk scores were lower in M0 versus M1 stage, although no study so far has reported necroptosis-associated lncRNAs as a risk factor for distant metastases in BC. However, similar risk scores of the older patients or those at higher tumor stages compared to their respective counterparts did not translate to similar survival prognoses, indicating that the combination of risk scores and clinicopathological features can better predict the prognosis of BC patients. The risk scores for C2 and C3 clusters were similar and significantly lower than that of C1 cluster, which was consistent with their respective survival prognoses. Patients with high immune scores, which corresponded to higher levels of TILs, had lower risk scores and a favorable prognosis. These findings are consistent with the current mainstream view on the role of TILs. Patients in the C2 and C3 clusters, and those with high immune scores were classified into the low-risk group, whereas the C1 cluster and low-immune scores comprised the high-risk group.

### 4.4. Cluster and Immune Infiltration Analysis

Studies show that increased infiltration of immune cells into tumor masses and high expression levels of PD-1/PD-L1 and CTLA-4 correlates with a favorable prognosis of BC [36, 37]. This suggests that lncRNAs associated with PD-L1 or CTLA-4 expression may be relevant to the immunotherapeutic response in BC, although there have been no reports so far. Consistent with our findings, the C3 cluster had significantly higher immune and ESTIMATE scores than C1. Interestingly, C2 had significantly lower immune, stromal, and ESTIMATE scores compared to C1, which did not correlate with the better prognosis in the former. The infiltration of memory B cells, CD8+ T cells, CD4+ T cells, follicular helper T cells, DCs, NK cells, and M1 macrophages was associated with anti-tumor effects, whereas Tregs and M2 macrophages promote tumor growth [38–40]. The anti-tumor immune cell types were significantly more enriched, whereas the M2 macrophages were less in the C3 cluster patients. Although C2 had fewer CD4 T cells compared to C1, it had a greater abundance of CD8+ T cells, NK cells, and plasma cells, which may explain the better survival prognosis in spite of a lower immune score. The *γΔ* T cells have both anti- and pro-tumorigenic functions [41] and were more abundant in the C3 clusters, although the role of this subset in BC has not been fully elucidated. A recent study showed that N1-type neutrophils inhibit tumor growth, whereas the N2-type neutrophils have a pro-cancer effect [42]. Infiltrating neutrophils were overall lower in the BC samples and did not differ between the clusters. Further studies are needed to explore the immune environment of breast tumors.

### 4.5. Immune Responsiveness of BC Patients

The low-risk group and the C3 and C2 clusters had similar immune cell infiltration. Immune checkpoint analysis further suggested that the low-risk group may be more sensitive to immunotherapy, and the results of drug sensitivity analysis can help screen potential therapeutic agents for low-risk patients. In summary, the C1 cluster and high-risk group correspond to immunologically “cold” tumors, whereas the “hot” tumors in C3, C2, and the low-risk group may be more sensitive to immunotherapy. In addition, the NR-lncRNAs are potential predictive markers of immune efficacy, and may help predict the immune efficacy of patients and improve individualized treatment.

### 4.6. GSEA for Risk Groups

Seo J et al. found that the E3 ubiquitin ligase negatively regulates the necroptosis proteins RIPK1 and RIPK3 through ubiquitination and lysosome-dependent degradation, which in turn inhibits necroptosis [43]. Furthermore, p53 regulates the NRF-miR-873 network, which inhibits the translation of RIPK1 and RIPK3 [44]. It is possible that E3 ubiquitin ligases have a similar function in BC and would therefore be a potential therapeutic target. Further studies are needed to explore its mechanism of action.

Some studies suggest that necroptosis of tumor cells recruits TILs and enhances the immune effect of it after releasing response by promoting the release of inflammatory mediators such as calreticulin, HMGB1, ATP, IL-6, and IL-33, which strengthens the immunotherapeutic effect by converting the cold tumors to hot [45]. However, little is known regarding necroptosis induction by lymphocytes and its bearing on the response to immunotherapy [46]. Lipid oxidation function was abundant in the low-risk group, which raises the possibility that the intra-tumoral cytotoxic lymphocytes increase the level of oxidized lipids in tumor cells, resulting in necroptosis and increased response to immunotherapy. Taken together, necroptosis and immunotherapy may act synergistically against tumor growth, which would be an interesting hypothesis.

## 5. Conclusion

The combination of necroptosis sensitizers and immune checkpoint inhibitors can improve the efficacy of immunotherapy in tumors with low necroptosis potential. However, further studies are needed to determine the degree of induction and necroptosis. Our findings regarding the cross-talk between necroptosis, tumor immune landscape, and prognosis may help in the design of individualized immunotherapy protocols to improve the survival outcomes of BC patients.

### 5.1. Limitations of our Study

Our data was retrieved from public databases and did not cover all cases in the relevant regions. Besides, the predictive performance of the model in the validation set was not completely satisfactory. Therefore, our findings have to be confirmed further through functional assays.

## Figures and Tables

**Table 1:** Clinical features of training and validation sets.

Variables	Training set (*n* =540)		Validation set (*n* =538)		*p*-value
	No.	%	No.	%	
Age					0.8017
< =60	295	54.36	299	55.58	
>60	245	45.37	239	44.42	
Gender					0.9514
Female	540	100	538	100	
Stage					0.895
I	88	16.30	93	17.29	
II	312	57.78	299	55.58	
III	119	22.04	125	23.23	
IV	10	1.85	9	1.67	
Unknown	11	2.04	12	2.23	
T stage					0.059
T1	142	26.30	136	25.28	
T2	324	60.00	297	55.20	
T3	54	10.00	83	15.43	
T4	20	3.70	19	3.53	
Unknown	0	0	3	0.56	
N stage					0.6715
N0	260	48.15	250	46.47	
N1	170	31.48	184	34.20	
N2	64	11.85	55	10.22	
N3	36	6.67	39	7.25	
Unknown	10	1.85	10	1.86	
M stage					0.6735
M0	449	83.15	448	83.27	
M1	12	2.22	9	1.67	
Unknown	79	14.63	81	15.06	

## Data Availability

The data used during the study are available at the TCGA (https://tcga-data.nci.nih.gov/tcga/) and the code is available from the corresponding author by request.

## Supplementary Materials

Supplementary Materials Figure S1: Differential expression analysis of 13 lncRNAs between tumor and normal samples. Figure S2: Heat map of 13 lncRNAs and clinicopathological factors. Figure S3: Differential expression analysis of 13 lncRNAs among C1, C2 and C3. Figure S4: Immune cells infiltration in C1, C2, and C3. Figure S5, S6: IC50 of anti-cancer drugs in high- and low-risk groups. Appendix 1: Sixty-seven necroptosis-associated lncRNAs. Appendix 2: Forty-seven immune checkpoint genes.
