# Supplementary material for: Necroptosis-Associated lncRNA Prognostic Model and Clustering Analysis: Prognosis Prediction and Tumor-Infiltrating Lymphocytes in Breast Cancer
Source: J Oncol. 2022 Apr 27;2022:7099930. doi: 10.1155/2022/7099930 (PMC9068297; doi:10.1155/2022/7099930)
Supplement: Supplementary Materials — Figure S1: Differential expression analysis of 13 lncRNAs between tumor and normal samples. Figure S2: Heat map of 13 lncRNAs and clinicopathological factors. Figure S3: Differential expression analysis of 13 lncRNAs among C1, C2 and C3. Figure S4: Immune cells infiltration in C1, C2, and C3. Figure S5, S6: IC50 of anti-cancer drugs in high- and low-risk groups. Appendix 1: Sixty-seven necroptosis-associated lncRNAs. Appendix 2: Forty-seven immune checkpoint genes. [file 7099930.f1.zip › Appendix 2 (1).pdf]

Immune checkpoint
ADORA2A
BTLA
BTNL2
C10orf54
CD160
CD200
CD200R1
CD244
CD27
CD274
CD276
CD28
CD40
CD40LG
CD44
CD48
CD70
CD80
CD86
CTLA4
HAVCR2
HHLA2
ICOS
ICOSLG
IDO1
IDO2
KIR3DL1
LAG3
LAIR1
LGALS9
NRP1
PDCD1
PDCD1LG2
TIGIT
TMIGD2
TNFRSF14
TNFRSF18
TNFRSF25
TNFRSF4
TNFRSF8
TNFRSF9
TNFSF14
TNFSF15

TNFSF18
TNFSF4
TNFSF9
VTCN1
