# Supplementary figures and images for: Necroptosis-Associated lncRNA Prognostic Model and Clustering Analysis: Prognosis Prediction and Tumor-Infiltrating Lymphocytes in Breast Cancer
Source: J Oncol. 2022 Apr 27;2022:7099930. doi: 10.1155/2022/7099930 (PMC9068297; doi:10.1155/2022/7099930)

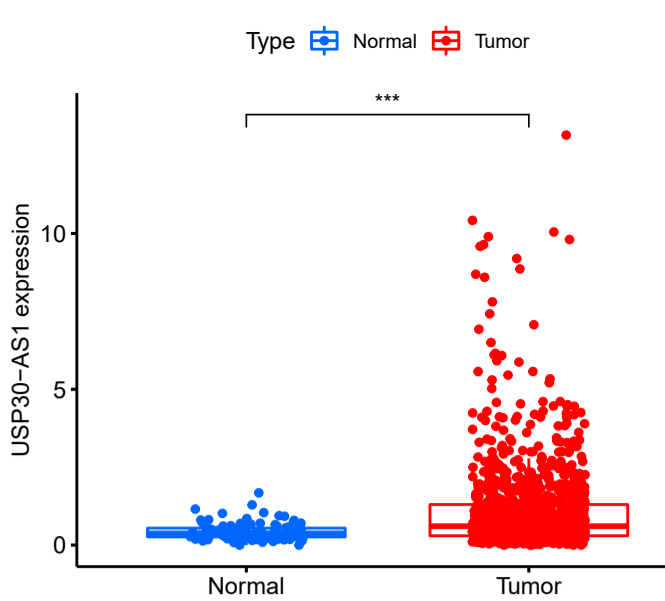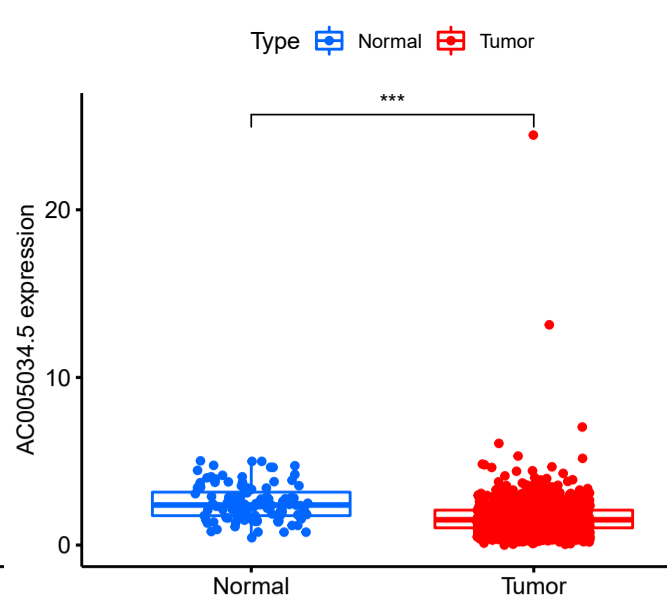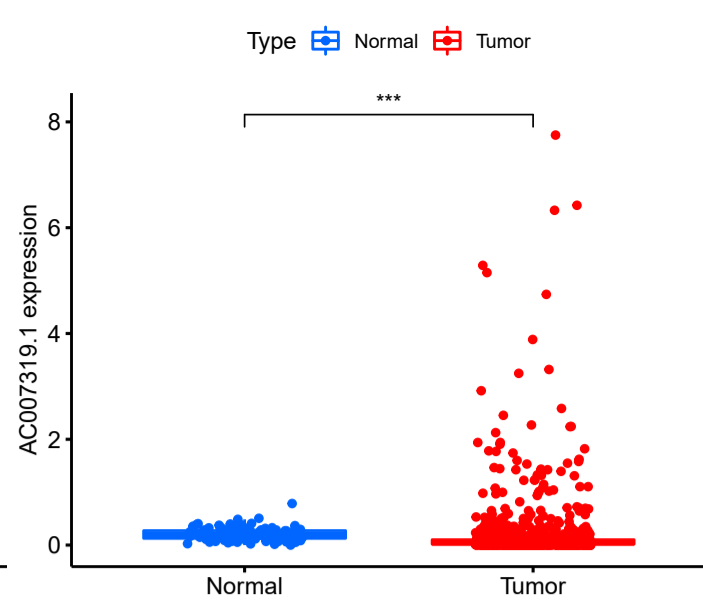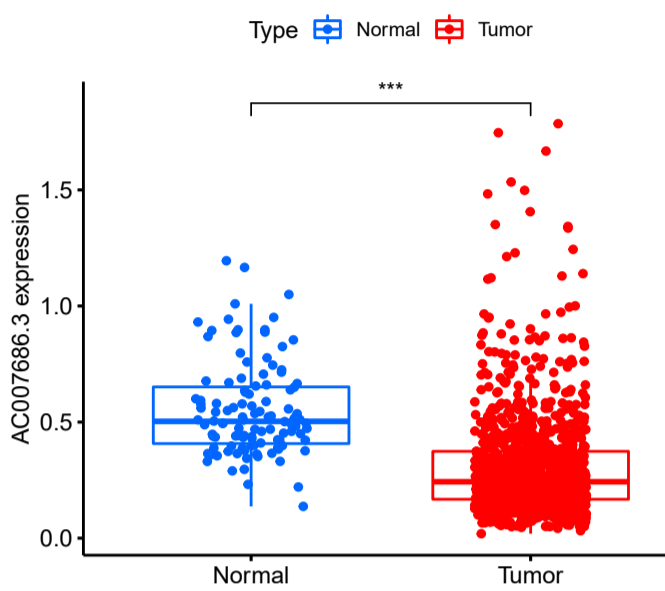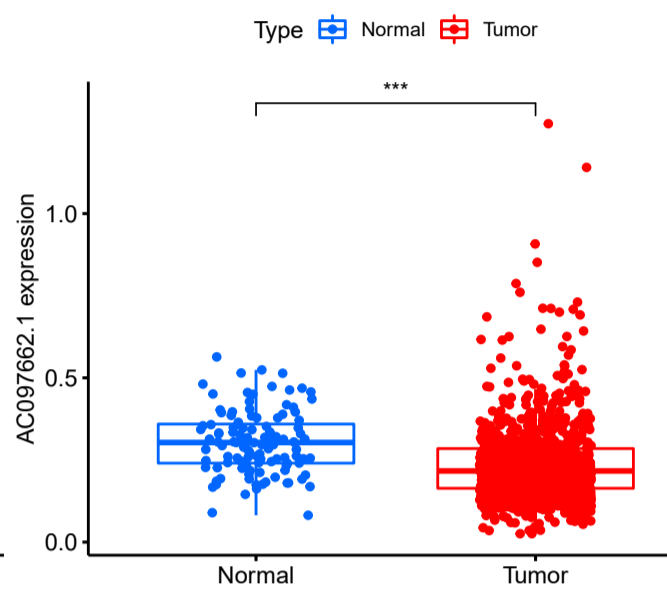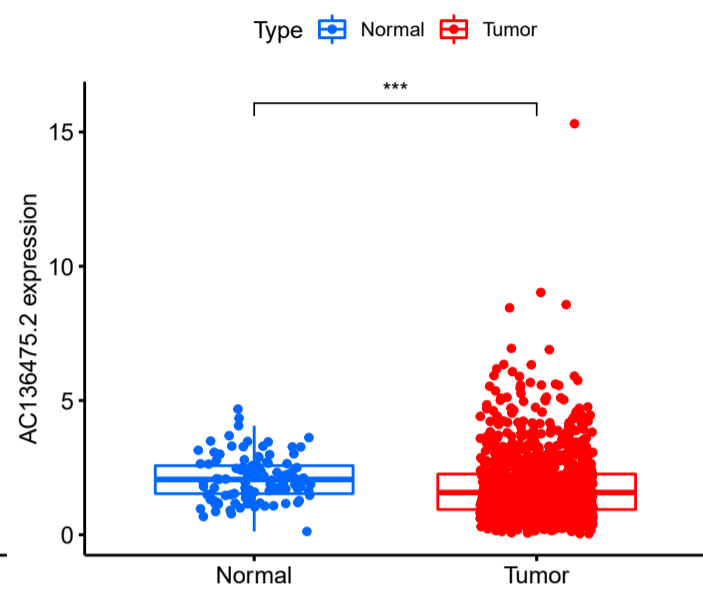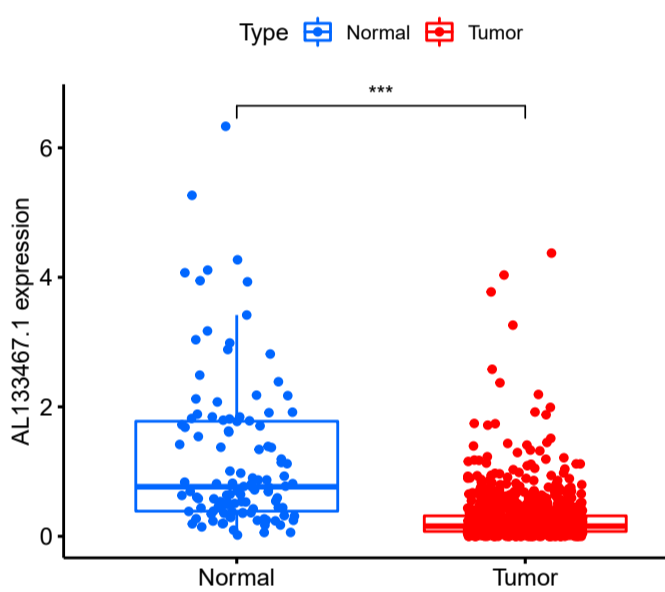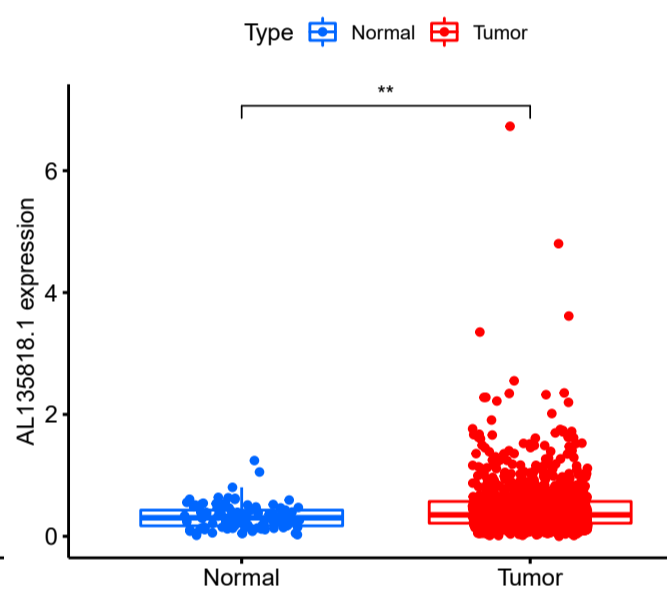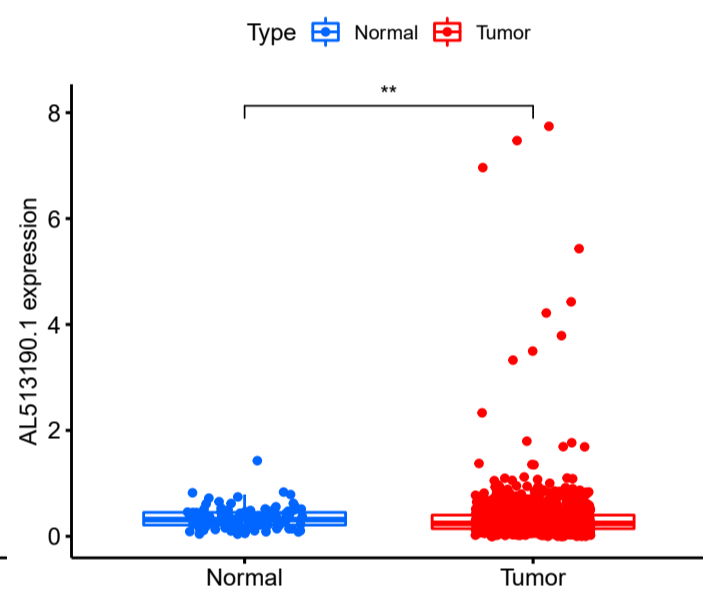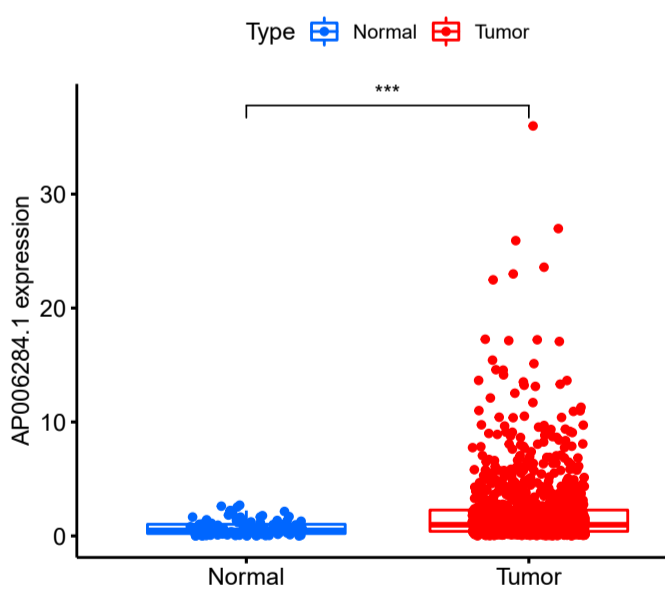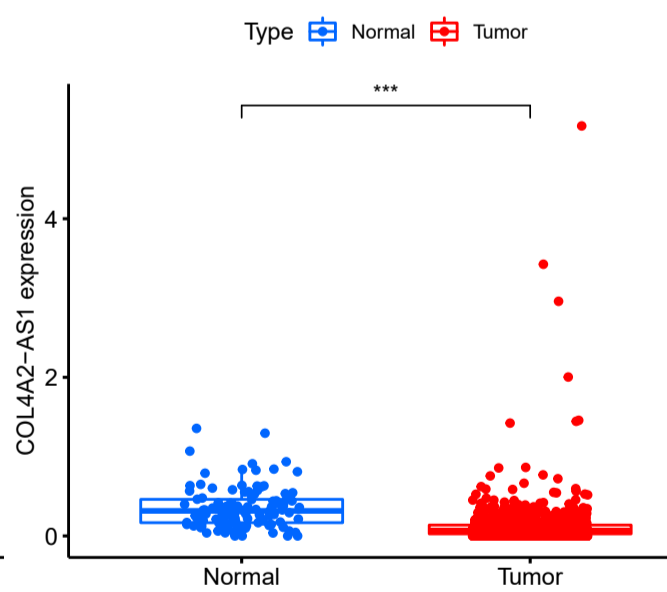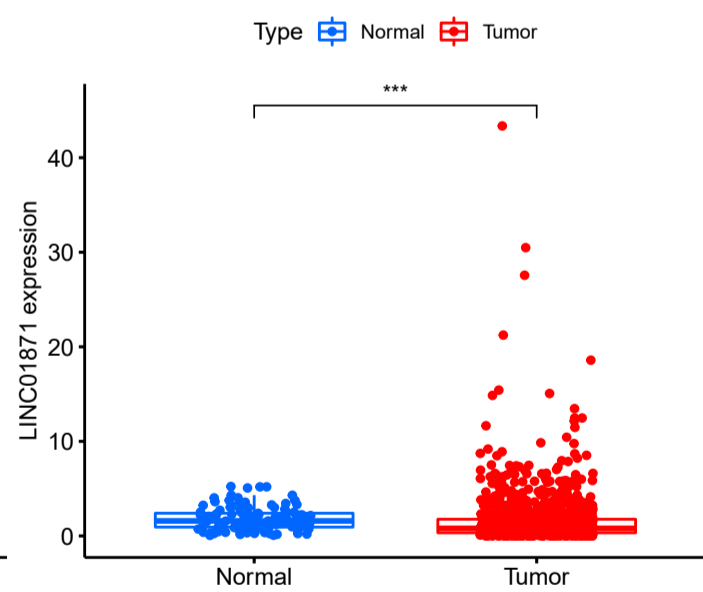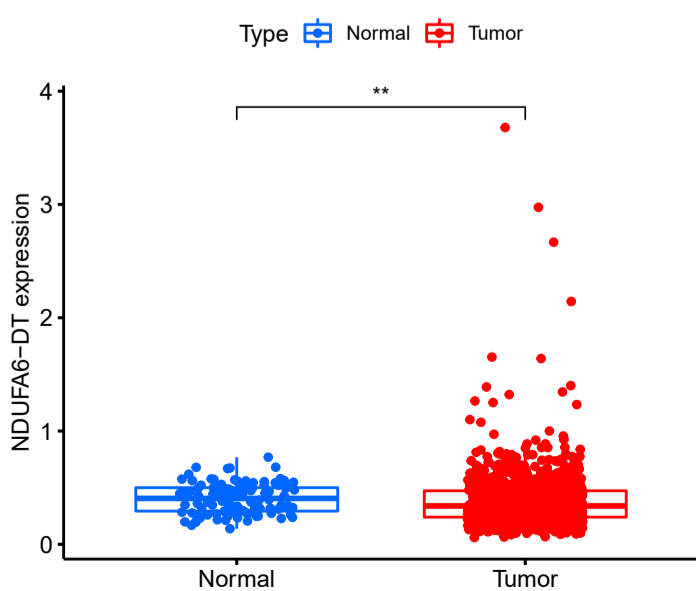

Supplement: Supplementary Materials — Figure S1: Differential expression analysis of 13 lncRNAs between tumor and normal samples. Figure S2: Heat map of 13 lncRNAs and clinicopathological factors. Figure S3: Differential expression analysis of 13 lncRNAs among C1, C2 and C3. Figure S4: Immune cells infiltration in C1, C2, and C3. Figure S5, S6: IC50 of anti-cancer drugs in high- and low-risk groups. Appendix 1: Sixty-seven necroptosis-associated lncRNAs. Appendix 2: Forty-seven immune checkpoint genes. [file 7099930.f1.zip › Figure-S1.pdf]

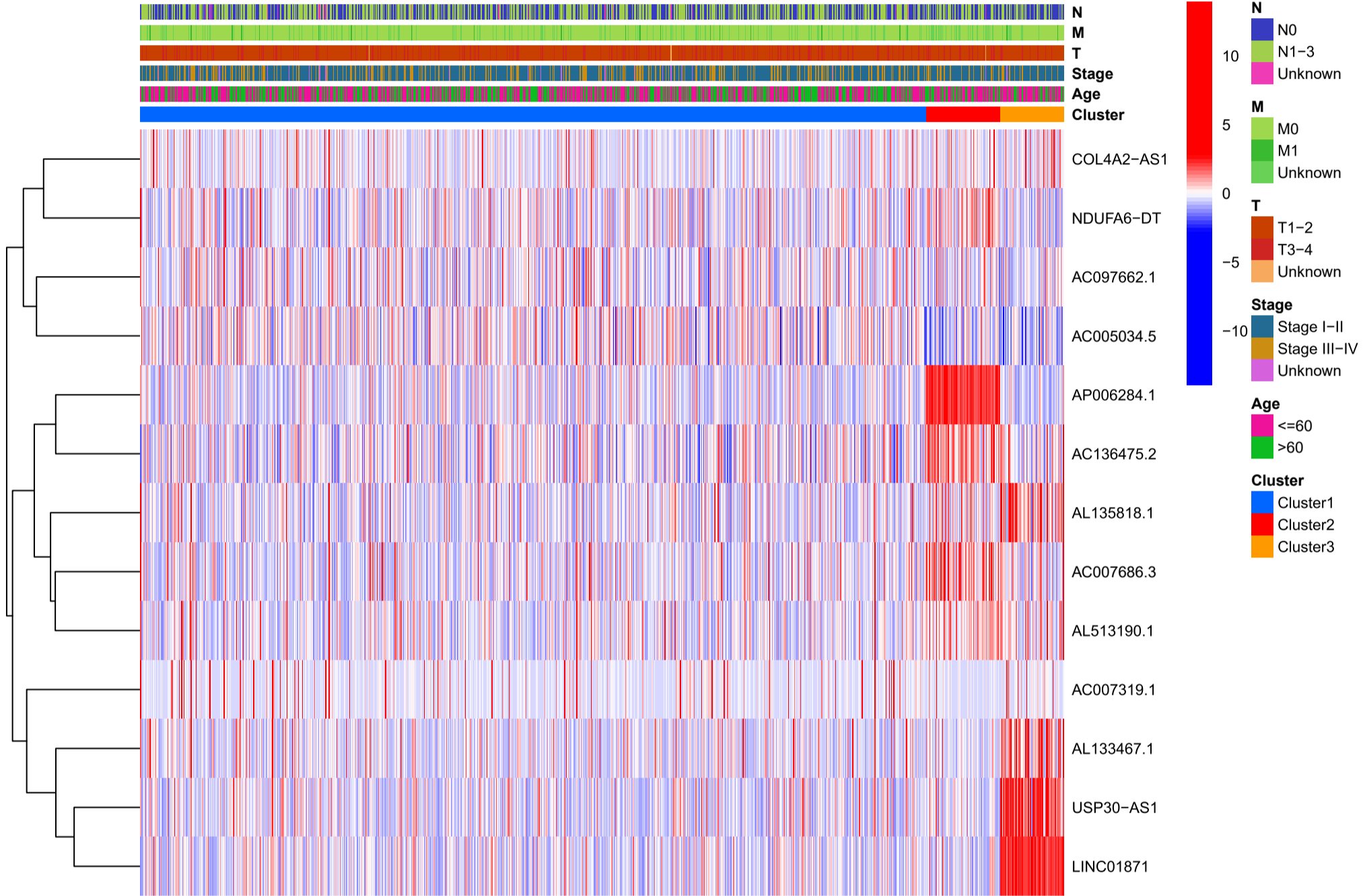

Supplement: Supplementary Materials — Figure S1: Differential expression analysis of 13 lncRNAs between tumor and normal samples. Figure S2: Heat map of 13 lncRNAs and clinicopathological factors. Figure S3: Differential expression analysis of 13 lncRNAs among C1, C2 and C3. Figure S4: Immune cells infiltration in C1, C2, and C3. Figure S5, S6: IC50 of anti-cancer drugs in high- and low-risk groups. Appendix 1: Sixty-seven necroptosis-associated lncRNAs. Appendix 2: Forty-seven immune checkpoint genes. [file 7099930.f1.zip › Figure-S2.pdf]

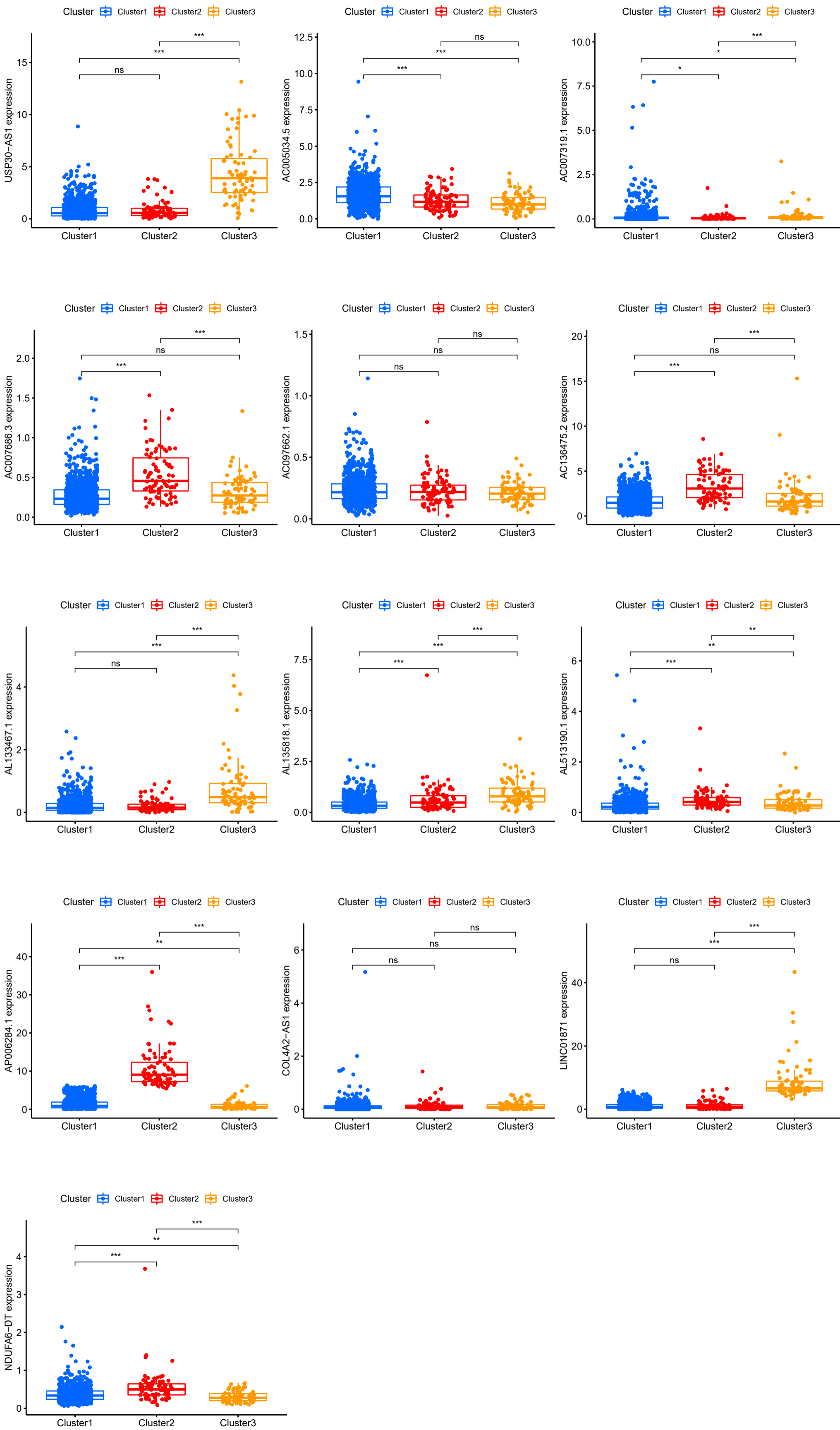

Supplement: Supplementary Materials — Figure S1: Differential expression analysis of 13 lncRNAs between tumor and normal samples. Figure S2: Heat map of 13 lncRNAs and clinicopathological factors. Figure S3: Differential expression analysis of 13 lncRNAs among C1, C2 and C3. Figure S4: Immune cells infiltration in C1, C2, and C3. Figure S5, S6: IC50 of anti-cancer drugs in high- and low-risk groups. Appendix 1: Sixty-seven necroptosis-associated lncRNAs. Appendix 2: Forty-seven immune checkpoint genes. [file 7099930.f1.zip › Figure-S3.pdf]

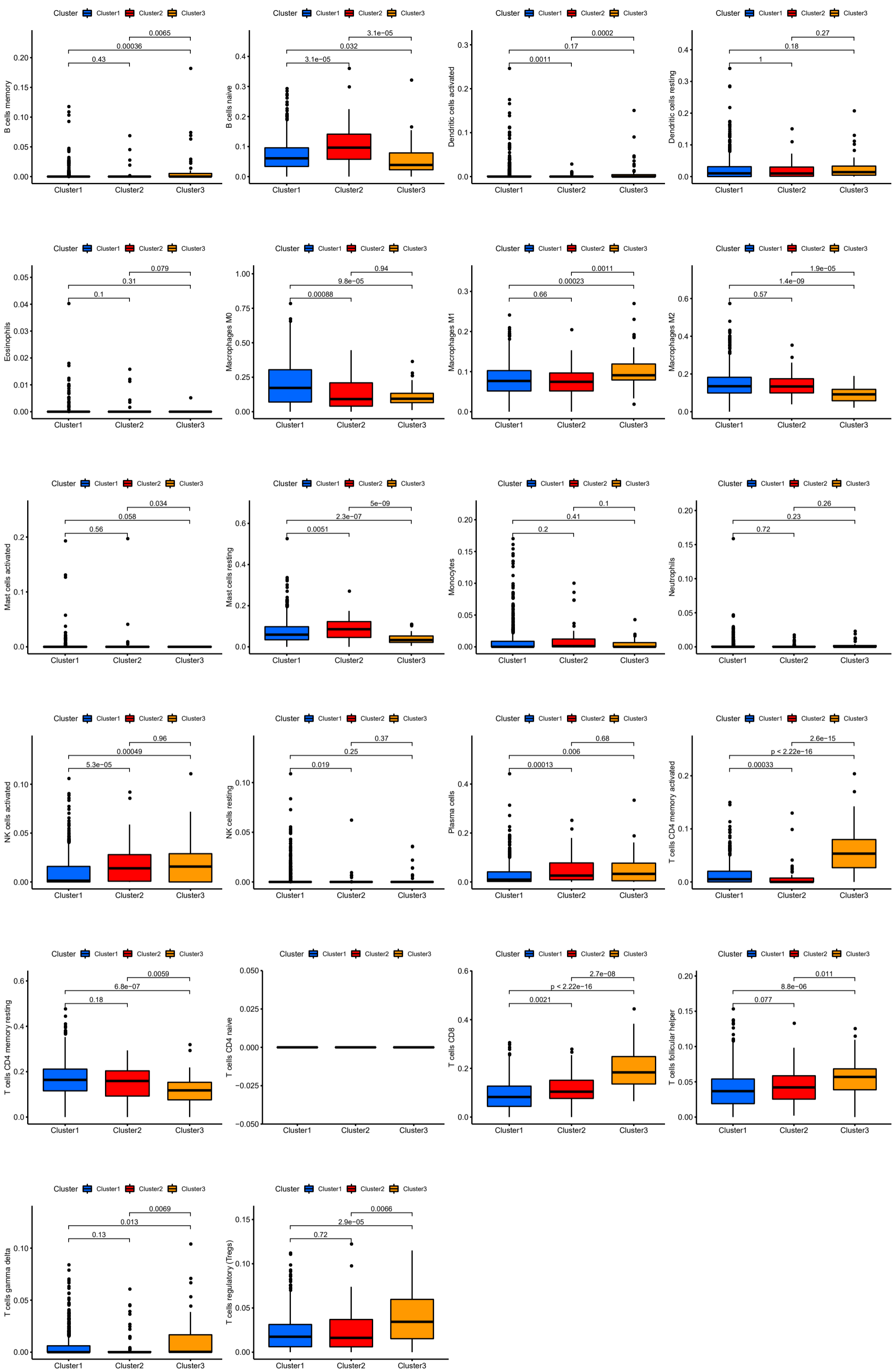

Supplement: Supplementary Materials — Figure S1: Differential expression analysis of 13 lncRNAs between tumor and normal samples. Figure S2: Heat map of 13 lncRNAs and clinicopathological factors. Figure S3: Differential expression analysis of 13 lncRNAs among C1, C2 and C3. Figure S4: Immune cells infiltration in C1, C2, and C3. Figure S5, S6: IC50 of anti-cancer drugs in high- and low-risk groups. Appendix 1: Sixty-seven necroptosis-associated lncRNAs. Appendix 2: Forty-seven immune checkpoint genes. [file 7099930.f1.zip › Figure-S4.pdf]

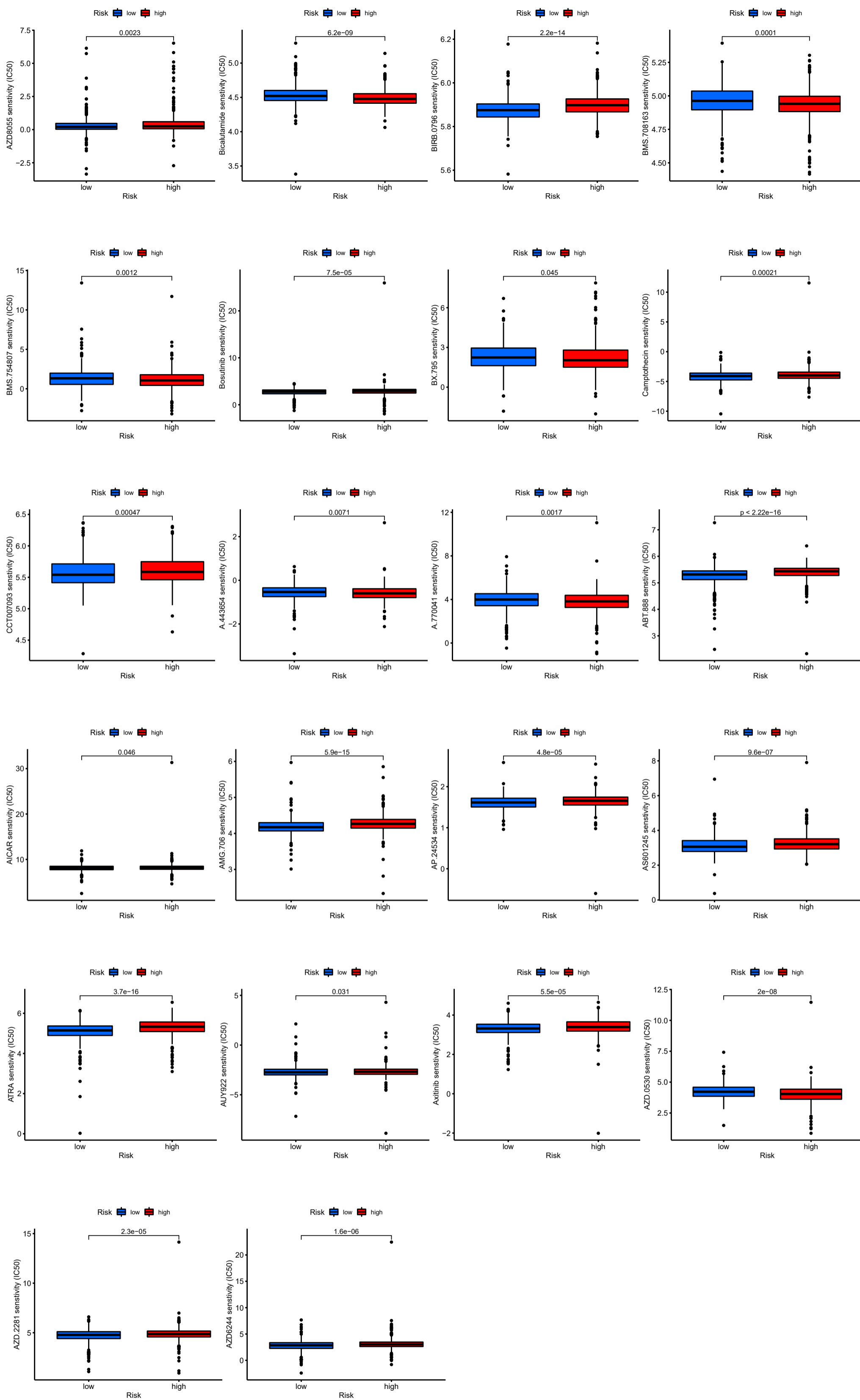

Supplement: Supplementary Materials — Figure S1: Differential expression analysis of 13 lncRNAs between tumor and normal samples. Figure S2: Heat map of 13 lncRNAs and clinicopathological factors. Figure S3: Differential expression analysis of 13 lncRNAs among C1, C2 and C3. Figure S4: Immune cells infiltration in C1, C2, and C3. Figure S5, S6: IC50 of anti-cancer drugs in high- and low-risk groups. Appendix 1: Sixty-seven necroptosis-associated lncRNAs. Appendix 2: Forty-seven immune checkpoint genes. [file 7099930.f1.zip › Figure-S5.pdf]

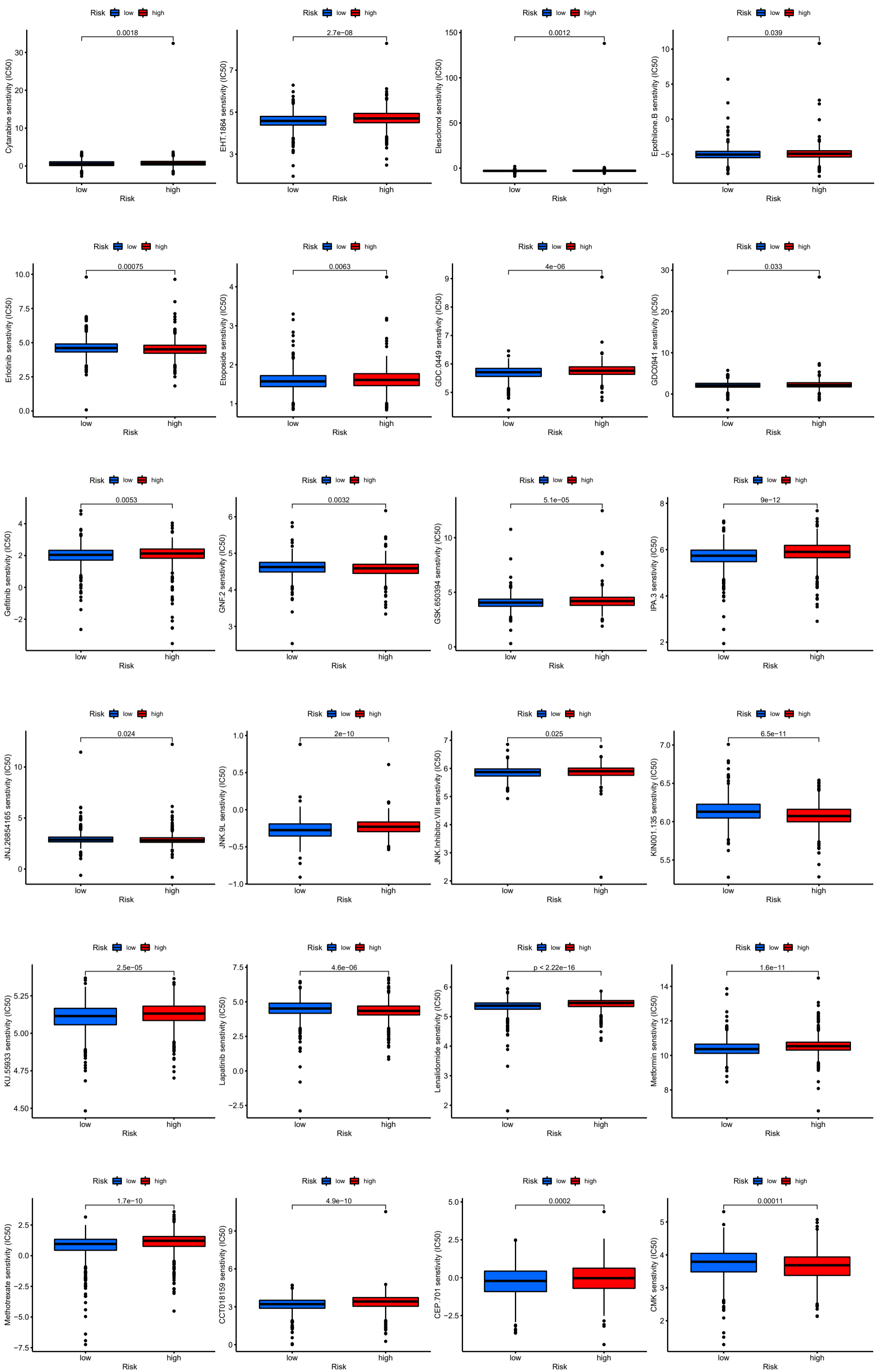

Supplement: Supplementary Materials — Figure S1: Differential expression analysis of 13 lncRNAs between tumor and normal samples. Figure S2: Heat map of 13 lncRNAs and clinicopathological factors. Figure S3: Differential expression analysis of 13 lncRNAs among C1, C2 and C3. Figure S4: Immune cells infiltration in C1, C2, and C3. Figure S5, S6: IC50 of anti-cancer drugs in high- and low-risk groups. Appendix 1: Sixty-seven necroptosis-associated lncRNAs. Appendix 2: Forty-seven immune checkpoint genes. [file 7099930.f1.zip › Figure-S6.pdf]
